# Urothelial carcinoembryonic antigen 1 score for early detection of prostate cancer and risk prediction

**DOI:** 10.1002/cam4.4629

**Published:** 2022-03-15

**Authors:** Youdong Lin, Guihua Liu, Chun Liu, Hui Xie, Xiaoxian Wang, Yudian Huang, Long Jin, Huidan Chen

**Affiliations:** ^1^ Department of Clinical Laboratory Medicine, Fujian Provincial Hospital Fujian Shengli Clinical Medical College of Fujian Medical University Fuzhou Fujian China; ^2^ Department of Children Health Care, Fujian Provincial Maternity and Children's Hospital Affiliated Hospital of Fujian Medical University Fuzhou Fujian Province China; ^3^ Department of urinary surgery Fujian Provincial Hospital Fuzhou Fujian China; ^4^ Department of Pathology Fujian Provincial Hospital Fuzhou Fujian China; ^5^ Department of urinary surgery Fuzhou NO. 1 Hospital Affiliated with Fujian Medical University Fuzhou Fujian China; ^6^ Department of Clinical Laboratory Medicine Fuzhou NO. 1 Hospital Affiliated with Fujian Medical University Fuzhou Fujian China; ^7^ Department of Pathology Fuzhou NO. 1 Hospital Affiliated with Fujian Medical University Fuzhou Fujian China

## Abstract

**Purpose:**

To improve early detection and risk prediction of prostate cancer, we incorporated the urine urothelial carcinoembryonic antigen 1 (UCA1) gene expression levels and the serum PSA level.

**Patients and Methods:**

In 897 patients the urine UCA1 gene expression was normalized and the performance of UCA1 score was evaluated by receiver operating characteristic (ROC) curve analysis, the Mann–Whitney *U* test, or decision curve analysis (DCA).

**Results:**

In the Shengli training cohort (*n* = 517), the area under ROC curve (AUC) was 0.880, 0.728, and 0.705 for detecting prostate cancer, D'Amico, and cancer of the prostate risk assessment (CAPRA), respectively. The UCA1 scores of benign patients were significantly lower than those of nonhigh‐risk prostate cancer patients (−2.63 vs. 0.16, *p* < 0.001; AUC 0.834). DCA yielded a better result with the UCA1 score compared to PSA. Combining PSA <4 with UCA1 score ≥−0.475, all nine of 62 patients were successfully diagnosed with prostate cancer and 70.97% of prostate biopsies were excluded. Using PSA ≥4 and UCA1 score ≥−3.47, 122 cancer patients were accurately diagnosed with a sensitivity of 0.992, while 102 prostate biopsies (22.42%) were excluded. Similar results were validated in the Fuzhou validation cohort (*n* = 380). In all patients of two cohort (*n* = 897), the UCA1 score was superior to PSA only for detection of clinically significant cancer (28% VS. 22%, *p* = 0.007) and detection of high‐risk cancer (25% VS. 19%, *p* = 0.009).

**Conclusion:**

The performance of the UCA1 score is superior to that of the existing PSA only in the detection and risk prediction of prostate cancer. Combination of the PSA level and the UCA1 score may significantly reduce the burden of prostate biopsy.

UCA1 score appears useful in detecting nonhigh‐risk (including very low‐, low‐, or intermediate‐risk) prostate cancer. Combination of the PSA level and the UCA1 score may significantly reduce the burden of prostate biopsy.

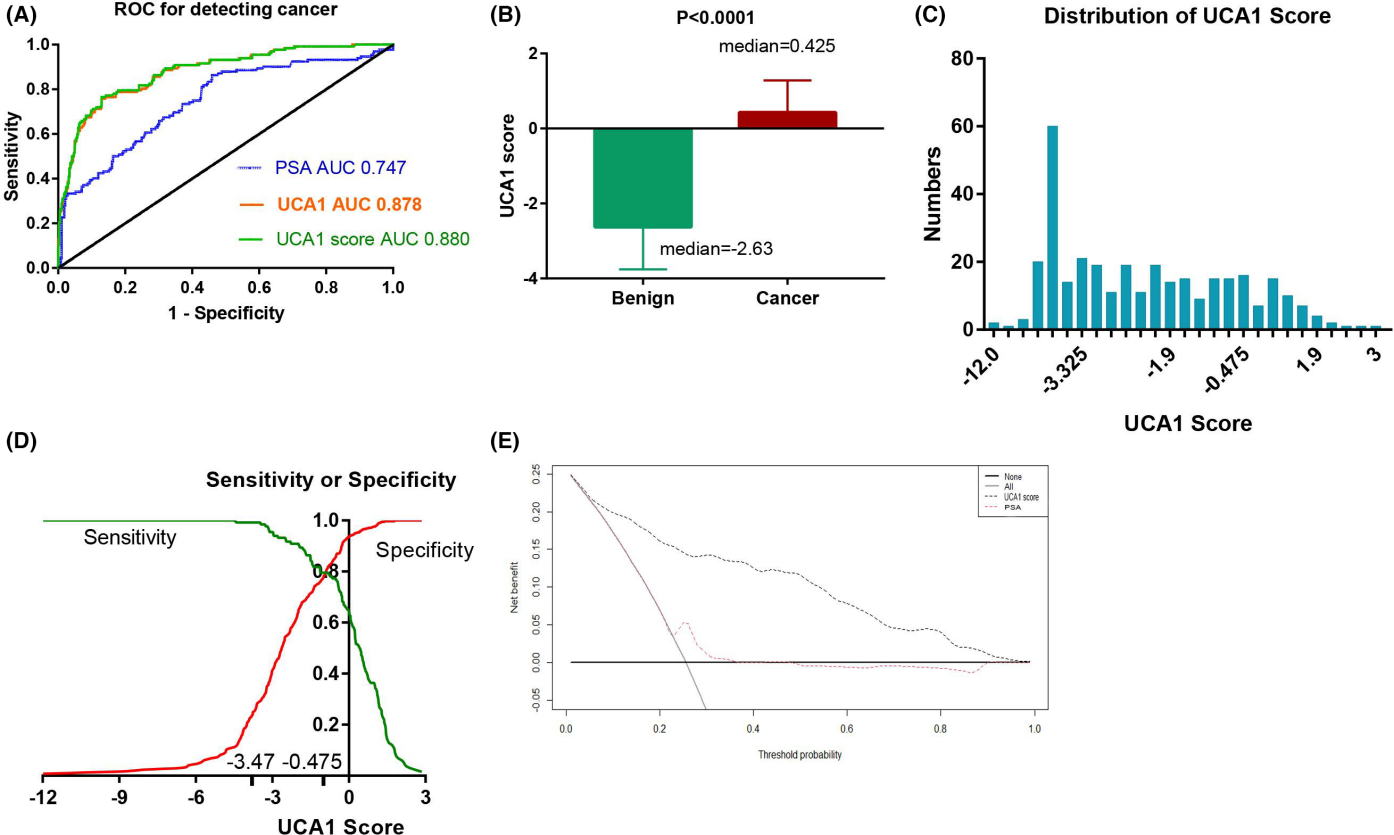

## 1 INTRODUCTION

Prostate‐specific antigen (PSA) is an important screening test tool for prostate cancer, which is used worldwide. However, the debate on the benefits of PSA for early prostate cancer detection continues. The 2012 U.S. Preventive Services Task Force (USPSTF) recommended against PSA‐based screening due to a high rate of false positive results, an excess of diagnostic biopsies, overdiagnosis, and overtreatment.^1^ Most major societies have updated and published guidelines.^2^, ^3^, ^4^, ^5^, ^6^ Some biomarkers for early prostate cancer detection such as PSA isoforms and prostate cancer3 (PCA3) are emerging. These biomarkers significantly decreased the number of diagnostic prostate biopsies^7^, ^8^, ^9^, ^10^, ^11^, ^12^ and have been incorporated into some guidelines, although their performance is not yet entirely established. Although imaging techniques such as MRI have made great advances in the detection of prostate cancer,^13^, ^14^, ^15^ noninvasive liquid biopsy markers are still of great significance. Therefore, we explored whether the UCA1 score can be used as a potential biomarker for prostate cancer detection or risk prediction.

Long noncoding RNA (lncRNA) UCA1, which is expressed as a proto‐oncogene and plays a key role in tumorigenesis and tumor development, is highly expressed in various tumor tissues and cell lines, including prostate cancer.^16^, ^17^, ^18^, ^19^ UCA1 promotes prostate cancer development by upregulating MYO6 expression through “sponging” miR‐143.^16^ UCA1 also regulates the growth and metastasis of prostate cancer by a competing endogenous RNA mechanism that regulates the miR‐204–CXCR4 axis.^17^ In bladder cancer, UCA1 increases ATF2 expression by binding miR‐204 and promotes cancer progression.^19^ Currently, prostate cancer risk stratification is widely used, including National Comprehensive Cancer Network (NCCN) high‐risk group (where the high‐risk group is defined as PSA ≥20, Gleason score ≥8, and/or ≥ stage T3), D'Amico,^20^ and CAPRA.^21^, ^22^ In this cohort, nonhigh‐risk cancer was defined as very low‐risk, low‐risk, or intermediate‐risk prostate cancer. Therefore, in this trial, we explored the performance of the UCA1 score for prostate cancer detection and risk stratification.

## 2 PATIENTS AND METHODS

### 2.1 Patients and study design

From June 2017 to July 2020, 517 patients scheduled for a diagnostic prostate biopsy at Fujian Shengli Clinical Medical College of Fujian Medical University were invited to participate (Shengli training cohort). Moreover, 380 patient samples were collected from the Fuzhou NO. 1 Hospital Affiliated with Fujian Medical University (Fuzhou validation cohort) between July 2019 and June 2021 (Figure S1). They were required to fulfill at least one of the following inclusion criteria: (1) PSA >10 ng/ml; (2) transrectal ultrasound (TRUS), computed tomography (CT), or magnetic resonance imaging (MRI) revealed suspicious lesions; (3) suspicious digital rectal examination (DRE); (4) PSA 4–10 ng/ml, free PSA/PSA >15%, or the PSA delta value was suspicious. Exclusion criteria were the following: (1) acute infection or fever; (2) hypertensive crisis; (3) period of cardiac insufficiency and decompensation; (4) diabetic unstable phase of serum glucose; (5) serious internal or external hemorrhoids; (6) perianal or rectal lesions; (7) a history of cancer; (8) with prior prostate resection; (9) with prostate biopsy within 6 months or a saturation biopsy at any time in the past. According to the NCCN Guidelines Version 2.2018 Prostate Cancer Early Detection^23^ and the Asian ethnicity of the patients, the indications of prostate biopsy in our cohort included the inclusion criteria described above, in addition to age and family history. As a prostate biopsy model for comparison, we only plused UCA1 scores as a pre‐biopsy work‐up of indications for biopsy. This trial was approved by the review boards of the two hospitals, and written informed consent was provided by the patients. The data that supports the findings of this study are available in the supplementary material of this article (Data S1 and S2).

### 2.2 Sample collection and assays

Serum PSA testing was performed using an electrochemical analyzer (COBAS E602, Roche Ltd, Germany) in accordance with the standard operating procedures of clinical specimens. Urine specimens for analysis were collected after DRE and before prostate biopsy according to SOP (standard operating procedure). Comparable to a protocol as for the PCA3 test reported in the janourly Clin Chem^24^, in our study urine samples from DRE to centrifugation for urine sediment separation must be completed within 2 h. All urine samples were centrifuged immediately after they were received by the laboratory staff. From each patient, two fresh 10‐ml urine specimens were collected in special tubes. The tubes were immediately centrifuged at 3000 *g* for 5 min, and the supernatant was discarded. To the remaining sediment, 3 ml of sterile physiological saline was added, and samples were centrifuged at 3000 *g* for 5 min. The supernatant was discarded and 0.7 ml of TRIzol RNA extraction reagent was immediately added to the sediment. The specimens were stored at −80°C. RNA was extracted in batches per week and reverse transcribed into cDNA, and then, the UCA1 and KLK2 genes were amplified by quantitative PCR using 18S rRNA as a housekeeping gene. The 18S rRNA, UCA1, and KLK2 primer sequences for qRT‐PCR are listed in Table S1. According to MIQE guidelines^25^, we performed quality control for RT‐qPCR and the coefficient of variation (CV) of intraassay or interassay is ≤10% in our study. The brief introduction is as follows: the concentration, purity, and integrity of RNA meet the requirements; RNA extraction, reverse transcription, and qPCR were performed according to the kit reagent instructions (TRANS Co., Beijing, China, https://WWW.transgen.com.cn). qPCR and data analysis were performed by using Roche LightCycler 480 (amplification protocol: 94°C, 30 s; 94°C,5 s and 57°C,30 s for 45 cycles); Water blank and reagent blank were set for negative control at each assay of amplification (the values were undetectable or greater than 43 cycles); As a housekeeping gene, the Cq values of 18S rRNA should be ≤30 cycles. Linearity, limit of detection (LOD), repeatability and reproducibility of qPCR are shown in Table S1. UCA1 gene expression (deta quantification cycle, △*C*_q_) = *C*_q UCA1 gene_ − *C*_q 18S rRNA gene_. KLK2 gene expression was shown as *C*_q KLK2 gene_. Urine UCA1 gene expression was normalized to urine KLK2 gene expression using the following formula: Normalized UCA1 gene expression = UCA1 gene expression × (median of *C*_q KLK2 gene_ of all samples divided by *C*_q KLK2 gene_). According to UCSC database (https://genome.ucsc.edu//gtex.html), KLK2 gene is mainly expressed in prostate cells in urine, so we used the median Cq value of all samples as a reference for each sample to relatively “normalize”.

### 2.3 Prostate biopsy and pathologic review

Prostate biopsies were performed under the guidance of B‐ultrasound by using a standard template.^3^ The standard template is a 12‐core biopsy scheme. As described in the NCCN guideline, Systematic prostate biopsy with TRUS guidance is the recommended technique for prostate biopsy. Commonly used scheme is the 12‐core biopsy scheme that includes a standard sextant and a lateral sextant scheme (lateral apex, lateral mid‐gland, and lateral base). All patients (*n* = 897) get 12‐core biopsy with TRUS guidance in this study. The size of the prostate was also measured by B‐ultrasound. The pathology report for each specimen was independently reviewed by a pathologist. Of each tissue specimen from the Fuzhou First Hospital, 10% was randomly selected to be reviewed by another senior pathologist from the Fujian Shengli Clinical Medical College of Fujian Medical University.

### 2.4 Statistical analysis

Sample size was estimated using the PmSamplesize R package,^26^ with predictor parameters (*p*) = 7, R2 = 0.9, and prevalence of prostate cancer = 8.65% in China.^27^ SPSS 19.0 and Prism7.0 were used for statistical analysis, and all tests were two‐tailed. *p* < 0.05 was considered to indicate statistical significance. Comparison of PSA levels or UCA1 scores between groups was performed using the Mann–Whitney *U* test. Specificity, sensitivity, positive predictive value (PPV), and negative predictive value (NPV) were calculated to evaluate the discriminatory ability of the UCA1 score. The AUC was calculated to evaluate diagnostic potential. The UCA1 score cutoff was defined as the point at which the sensitivity and specificity were optimal or the sensitivity was close to 1.0 plus the specificity by using the ROC was the highest. R software (version 3.6.1) was used for DCA. A Chi‐square test was used to compare percentages between UCA1 score and PSA.

## 3 RESULTS

### 3.1 Study population and UCA1 score calculation

The Shengli training cohort originally included 560 patients. Urine samples were collected from 548 patients. Metastases were confirmed in 19 of these patients and insufficient tissue samples were available from 12 patients, resulting in 517 patients with a valid UCA1 score (Figure S1). Finally, 132 of the 517 patients were pathologically diagnosed with prostate cancer. Among 380 patients in the Fuzhou validation cohort, 91 patients were diagnosed with cancer based on pathological assessment. Patient characteristics are presented in Table 1. Pearson correlation analysis demonstrated that the relationship between the KLK2 gene expression (KLK2 Cq value) and UCA1 gene expression (△Cq Value) showed correlation in the Shengli training cohort (*n* = 517, correlation coefficient = 0.99, *p* = 0.024) and Fuzhou validation cohort (*n* = 380, correlation coefficient = 0.99, *p* = 0.025) respectively, this proved that the UCA1 expression is related to the prostate cells in the urine sediment. The UCA1 score was calculated by binary logistic regression based on urine UCA1 gene expression and serum PSA levels: UCA1 score = 3.591 + 0.000475 × PSA value −0.375 × normalized UCA1 gene expression.

**TABLE 1:** Patients characteristics

Characteristic	Shengli training cohort (*n* = 517)			Fu zhou validation cohort (*n* = 380)			Patients population (*n* = 897)
	Benign	Cancer	*p* ^a^	Benign	Cancer	*p* ^a^	
Patients	385	132		289	91		897
Age, median, years	71	72	0.142	71	71	0.924	71
PSA, median, ng/ml	6.63	16.58	<0.0001	6.6	13.25	<0.0001	7.9
UCA1 score, median	−2.63	0.43	<0.0001	−2.44	0.56	<0.0001	−1.98
T‐stage							
T0/T1		30			17		47
T2		28			19		47
T3		35			24		59
T4		39			31		70
Gleason score							
≦6		17			11		28
7		47			29		76
8		35			29		64
≧9		33			22		55
NCCN risk group							
Nonhigh risk		30			19		49
High risk		102			72		174
D'Amico risk group							
LR/IR		24			19		43
HR		108			72		180
CAPRA risk group							
LR/IR		50			24		74
HR		82			67		149

*Note:* Nonhigh risk, including very low, low, or intermediate risk.

Abbreviations: LR, low risk; IR, intermediate risk; HR, high risk; CAPRA, cancer of the prostate risk assessment.

^a^ Mann–Whitney *U* test.

### 3.2 UCA1 score performance characteristics in the Shengli training cohort

#### 3.2.1 UCA1 score and prostate cancer detection

**FIGURE 1:**
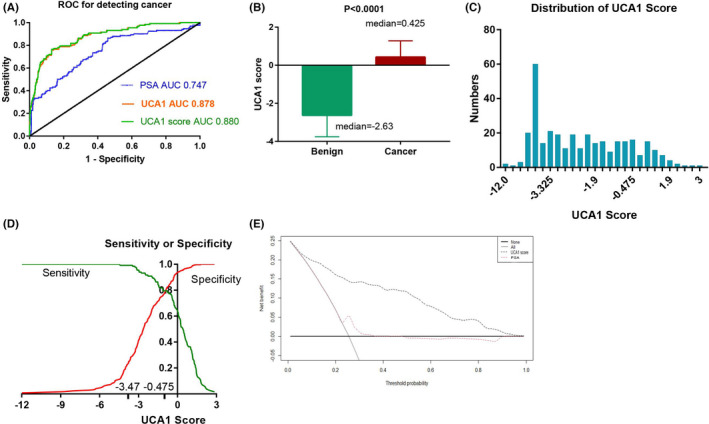
Performance characteristics of the UCA1 score in the Shengli training cohort. (A) ROC for detecting prostate cancer (benign vs. cancer). (B) UCA1 score of benign and cancer patients. (C) Histogram showing the distribution of predicted risks. (D) Decreased sensitivity and increased specificity are observed for increasing risk thresholds for the UCA1 score. (E) Decision curve analysis demonstrated a net clinical benefit of performing biopsy on patients. Abbreviations: ROC, receiver operating characteristic; PSA, prostate‐specific antigen; UCA1, urothelial carcinoembryonic antigen 1; AUC, area under the ROC curve

The UCA1 score was calculated for 517 samples. Binary logistic regression and ROC curve analyses were performed to calculate their ability to correctly detect cancer. The UCA1 score performed well in discriminating cancer from benign tissues. The AUC was 0.880 (95% confidence interval [CI], 0.846–0.914), which was significantly higher than that of serum PSA alone (AUC 0.747, 95% CI, 0.697–0.797) and similar to that of the urine UCA1 test only (AUC 0.877, 95% CI, 0.843–0.912) (Figure 1A).

The median UCA1 score for prostate cancer patients was 0.425 compared to −2.63 for benign patients (Figure 1B). The UCA1 scores of prostate cancer patients were significantly higher than those of benign patients. From Figure 1C, we can see that most of the patients in this cohort were benign patients. Based on the recommended UCA1 score threshold of ≥−0.475 (sensitivity and specificity were optimal), 150 of 517 patients were identified with prostate cancer (Figure 1D, Table 2). As cutoff value, the threshold had optimal sensitivity (0.765) and specificity (0.870). The number needed to test (NNT) to identify one prostate cancer patient at cutoff of −0.475 was approximately two individuals; if the UCA1 score was not used, four prostate biopsies were needed. The PPV was 0.67 and the NPV was 0.92 for individuals correctly detected as benign patients. Similarly, at a UCA1 score threshold of ≥−3.47 (sensitivity close to 1.0 plus the highest specificity), sensitivity and specificity were 0.992 and 0.294, respectively, and the NPV was 0.99 (Table 2).

**TABLE 2:** Performance characteristics of UCA1 score for detecting prostate cancer in the Shengli training cohort

Patients	Performance characteristics	UCA1 score ≥			
		−12	−3.47	−0.475	3
Benign vs. cancer	Sensitivity	1	0.992	0.765	0
	Specificity	0	0.294	0.870	1
	TP	132	131	101	0
	TN	0	113	335	385
	FP	385	272	49	0
	FN	0	1	31	132
	NNT	3.92	3.08	1.49	
	PPV	0.26	0.33	0.67	NaN
	NPV	NaN	0.99	0.92	0.74
Benign vs. nonhigh‐risk prostate cancer	Sensitivity	1		0.767	0
	Specificity	0		0.870	1
	TP	30		23	0
	TN	0		335	385
	FP	385		49	0
	FN	0		7	30
	NNT	13.8		3.13	
	PPV	0.072		0.32	NaN
	NPV	NaN		0.98	0.93

*Note:* FN, FP, TN, and TP are reported for a given cutoff value on the UCA1 score. NNT = (TP + FP)/TP.

Abbreviations: TP, true positive; TN, true negative; FP,false positive; FN, false negative; NaN, not a number; NNT, number needed to test; NPV, negative predictive value; PPV, positive predictive value.

By using DCA methodology, the clinical impact of the UCA1 score model to identify patients for prostate biopsy was observed at a probability threshold of approximately ≥0.05 (Figure 1E); maximal utility occurred at approximately 0.4. Compared with serum PSA, UCA1 scores had a better net benefit in prostate biopsy decision‐making, across the range of probability thresholds.

### 3.3 UCA1 score and nonhigh‐risk prostate cancer

**FIGURE 2:**
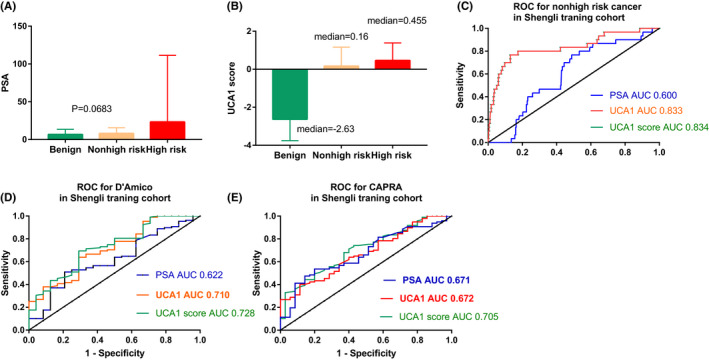
UCA1 score/PSA and nonhigh‐risk prostate cancer, D'Amico, and CAPRA. (A) PSA and nonhigh‐risk and high‐risk prostate cancer. (B) UCA1 score and nonhigh‐risk and high‐risk prostate cancer. (C) ROC curve for nonhigh‐risk cancer (nonhigh‐risk vs. high‐risk). (D) ROC curve for D'Amico (LR/IR vs. HR). (E) ROC curve for CAPRA (LR/IR vs. HR). Abbreviations: ROC, receiver operating characteristic; PSA, prostate‐specific antigen; UCA1, urothelial carcinoembryonic antigen 1; AUC, area under the ROC curve; LR, low risk; IR, intermediate risk; HR, high risk; CAPRA, cancer of the prostate risk assessment. Nonhigh‐risk includes very low‐, low‐, or intermediate risk

In the Shengli training cohort, PSA showed no difference between patients with nonhigh‐risk prostate cancer and benign patients (median 8.07 vs. 6.63, *p* = 0.0683, Figure 2A). The UCA1 score of high‐risk prostate cancer patients is dramatically higher than that of benign patients (median 0.455 vs. −2.630, *p* < 0.0001, Figure 2B). Notably, the UCA1 score of nonhigh‐risk prostate cancer patients was obviously higher than that of benign patients (median 0.160 vs. −2.630, *p* < 0.0001, Figure 2B). Furthermore, the UCA1 score performed well in distinguishing between nonhigh‐risk prostate cancer patients and benign patients, with an AUC value of 0.834 (Figure 2C). At a cutoff value of −0.475, 23 nonhigh‐risk prostate cancer patients of 415 patients were detected with optimal sensitivity (0.767) and specificity (0.870, Table 2). If the UCA1 score was used, the NNT decreased dramatically from 14 to three individuals and the NPV increased to 0.98 of individuals correctly detected as benign patients.

### 3.4 UCA1 score and D'Amico and CAPRA

For discriminating D'Amico high‐risk from low‐ and intermediate‐risk prostate cancer patients, the UCA1 score showed some usefulness. The AUC was 0.728 (95% CI, 0.617–0.838), which was better than that of serum PSA alone (AUC 0.622, 95% CI, 0.503–0.741) or the urine UCA1 test alone (AUC 0.710, 95% CI, 0.597–0.823) (Figure 2D). Similarly, to discriminate CAPRA high‐risk from low‐ and intermediate‐risk prostate cancer patients, the AUC of the UCA1 score of 0.705 (95% CI, 0.609–0.800) was a bit better than that of serum PSA alone (AUC 0.671, 95% CI, 0.572–0.771) or the urine UCA1 test alone (AUC 0.672, 95% CI, 0.574–0.771) (Figure 2E).

### 3.5 The UCA1 score assists prostate biopsy in the Shengli training cohort

First, we selected a UCA1 score cutoff of −0.475 and then combined the UCA1 score with PSA and other high‐risk biopsy criteria to simulate biopsy decisions for this cohort.

In patients with PSA <4, combined with UCA1 scores ≥−0.475, nine of 62 patients were successfully diagnosed with prostate cancer (Table 3). The number of patients recommended for biopsy was sharply reduced from 62 to 18, a reduction of 70.97%.

**TABLE 3:** UCA1 score assist prostate biopsy at different cutoff value in the Shengli training cohort

	PSA			
	<4	≥4 to <10	10–20	>20
Patients (*n* = 517)	62	230	120	105
Benign (*n* = 385)	53	199	84	49
UCA1 score <−0.475 (TN)	44	178	73	40
UCA1 score ≥−0.475 (FP)	9	21	11	9
Cancer (*n* = 132)	9	31	36	56
UCA1 score <−0.475 (FN)	0	6	10	15
UCA1 score ≥−0.475 (TP)	9	25	26	41
Recommend biopsy	18	46	37	50
Biopsy redution (*n*, %)	44 (70.97)	184 (80.00)	83 (69.17)	65 (61.90)
Missed diagnosis (*n*, %)	0 (0)	6 (2.61)	10 (8.33)	15 (14.29)
Benign (*n* = 385)	53	199	84	49
UCA1 score <−3.47 (TN)	12	61	24	16
UCA1 score ≥−3.47 (FP)	41	138	60	33
Cancer (*n* = 132)	9	31	36	56
UCA1 score <−3.47 (FN)	0	1	0	0
UCA1 score ≥−3.47 (TP)	9	30	36	56
Recommend biopsy	50	168	96	89
Biopsy redution (*n*, %)	12 (19.35)	62 (26.97)	24 (20.00)	16(15.24)
Missed diagnosis (*n*, %)	0 (0)	1 (0.43)	0 (0)	0 (0)

Abbreviations: TP, true positive; TN, true negative; FP, false positive; FN, false negative.

Similarly, when the PSA had a value of 4 ≤ PSA < 10, 10 ≤ PSA < 20, or PSA ≥20, the recommended number of biopsies decreased dramatically to 46, 37, or 50, respectively. However, the rate of missed diagnosis increased (6/230, 2.61%; 10/120, 8.33%, 15/105, 14.29%).

To avoid missed diagnosis, another UCA1 score cutoff of ≥−3.47 was applied for biopsy decision‐making. Using PSA ≥4 and UCA1 score ≥−3.47, 122 cancer cases were accurately detected in 455 patients with a sensitivity of 0.992 (only one cancer patient was missed) and a specificity of 0.294. Meanwhile, 102 prostate biopsies (22.42%) were avoided (Table 3).

### 3.6 UCA1 score performance characteristics in the Fuzhou validation cohort

**FIGURE 3:**
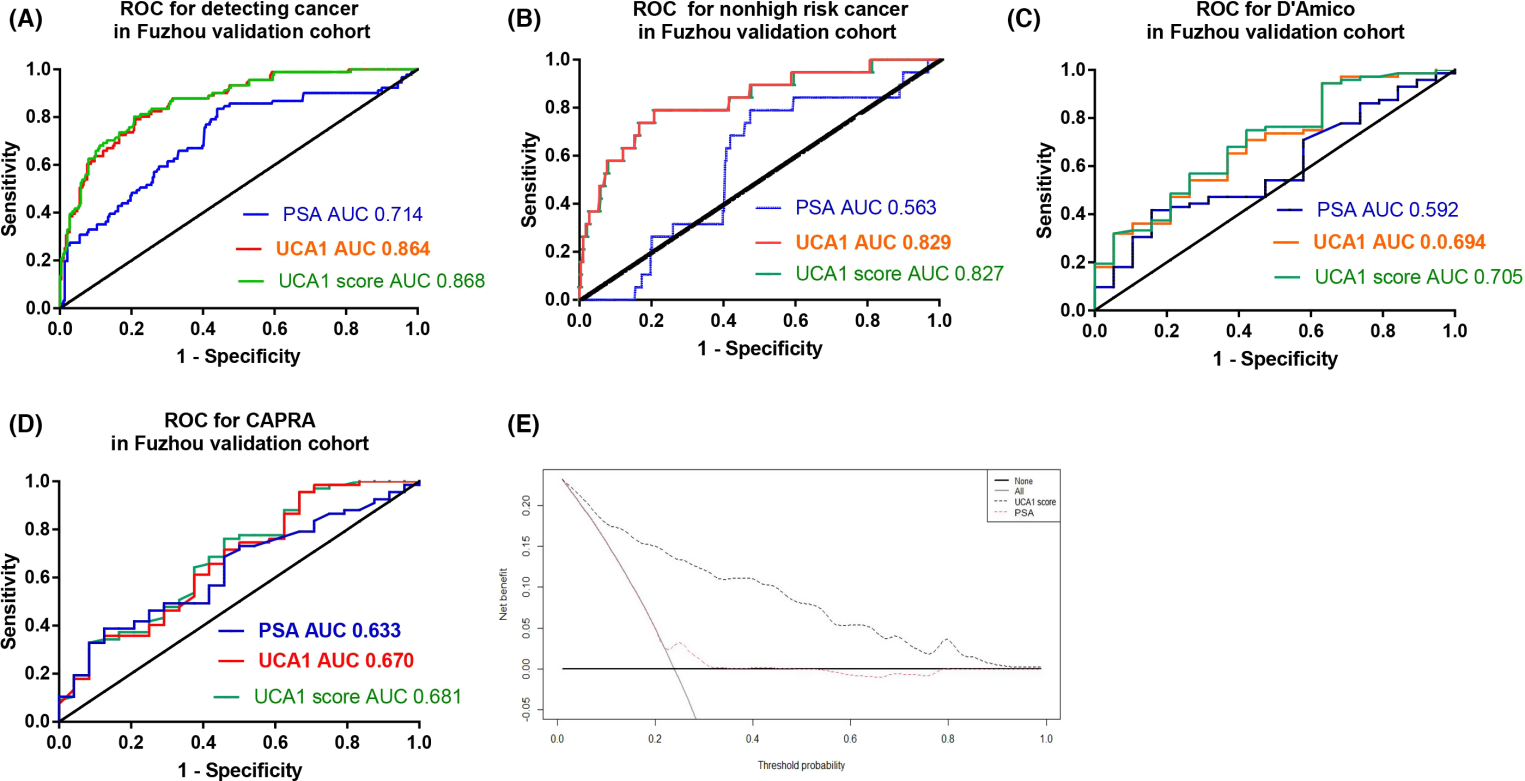
Performance characteristics of the UCA1 score in the Fuzhou validation cohort. (A) ROC for detecting prostate cancer (benign vs. cancer). (B) ROC curve for nonhigh‐risk cancer (nonhigh‐risk vs. high‐risk). (C) ROC curve for D'Amico (LR/IR vs. HR). (D) ROC curve for CAPRA (LR/IR vs. HR). (E) Decision curve analysis demonstrated a net clinical benefit of performing biopsy on patients. Abbreviations: ROC, receiver operating characteristic; PSA, prostate‐specific antigen; UCA1, urothelial carcinoembryonic antigen 1; AUC, area under the ROC curve; CAPRA, cancer of the prostate risk assessment. Nonhigh‐risk includes very low‐, low‐, or intermediate risk

In the Fuzhou validation cohort, the UCA1 score also performed well in the detection of prostate cancer, nonhigh‐risk prostate cancer, D'Amico, and CAPRA. The AUCs were 0.868, 0.827, 0.705, and 0.681, respectively (Figure 3A–D).

At a cutoff value of UCA1 score ≥−0.475, 112 of 380 patients were diagnosed with prostate cancer with a sensitivity of 0.714 and a specificity of 0.837 (Table S2). The NNT decreased from four to two patients needed for prostate biopsy if the UCA1 score was not used.

Similarly, at a cutoff value of UCA1 score ≥−3.47, all 91 cancer patients were diagnosed with a sensitivity of 1.0 and an NPV of 1.0.

As in the Shengli training cohort, in the Fuzhou validation cohort, DCA also demonstrated that UCA1 scores had a better net benefit in prostate biopsy decision‐ making than PSA (Figure 3E).

### 3.7 The UCA1 score assists prostate biopsy in the Fuzhou validation cohort

By using PSA <4 and UCA1 score ≥−0.475, all nine cancer patients were identified with a false negative (FN) value of 0 (Table S3). The recommended number of biopsies decreased sharply from 52 to 20.

When PSA ≥4 combined with UCA1 score ≥−3.47 was applied, all 82 of 91 cancer patients were correctly diagnosed with an FN value of 0 (Table S3).

### 3.8 The UCA1 score assists prostate biopsy in all patients

If UCA1 score had been used to assist biopsy in all patients of two cohort (*n* = 897), 197 patients could have been avoided. Cinically significant cancer (Gleason score ≥7)^28^ was diagnosed in 193 of 700 patients (28%) by using UCA1 score, as compared with 195 of 897 patients (22%) by using PSA only, a difference of 6 percentage points (95% CI, 2 to 10) (Table S4 and Figure S2). Because the lower boundary of the two‐sided 95% CI was greater than 0 percentage points,^26^, ^27^ the UCA1 score was deemed superior to PSA only for detection of clinically significant cancer (*p* = 0.007). For detecting clinically insignificant cancer (Gleason score 6), the difference between UCA1 score and PSA only was 1 percentage point (95% CI, 0 to 3), the UCA1 score was also superior to PSA only. For detecting group 3 + 4 + 5 of WHO grade groups cancer (Gleason score ≥4 + 3), the difference between UCA1 score and PSA only was 6 percentage point (95% CI, 1 to 9; *p* = 0.015), the UCA1 score was also superior to PSA only (Table S4).

Similarly, for detecting nonhigh‐risk, the difference was 2 percentage point (95% CI, −1 to 4) (Table S4). The lower boundary of the two‐sided 95% CI was greater than −4 percentage points^28^, ^29^, therefore the UCA1 score was noninferior to PSA only. For detecting high‐risk prostate cancer, the difference was 6 percentage point (95% CI, 1 to 10; *p* = 0.009). The UCA1 score (25%) was superior to PSA only (19%) (Table S4 and Figure S2).

We analyzed patients without elevated PSA (<4) by ROC and found that UCA1 score had a significantly higher AUC (0.977) than PSA only (AUC 0.693) (Figure S3) without missed diagnosis of prostate cancer.

## 4 DISCUSSION

Many reports of serum PSA screening for prostate cancer show a false positive rate of up to two‐thirds, resulting in an excessive burden of prostate biopsy.^30^, ^31^ As is well known, prostate biopsy is a huge burden on both patients and health resources, especially in the era of COVID‐19. The 2012 USPSTF recommendations highlight the limitations of PSA for prostate cancer screening and stress individualized diagnosis strategies and assessments of personal risk as future directions.^32^ Liquid tumor biopsy has been developing rapidly in recent years. In particular, urine contains exfoliated cells from the prostate gland and is easy to collect; so, a noninvasive genetic test for urine sediment has a good application prospect in the diagnosis and treatment monitoring of prostate cancer patients. According to the NCCN Guidelines Updates of 2018, PCA3 >35 can be used as a biomarker for early detection of prostate cancer.^33^ UCA1 is an lncRNA similar to PCA3, and the mechanism of promoting the occurrence and development of prostate cancer has been widely explored.^16^, ^17^, ^18^ Therefore, we assessed the performance of the UCA1 score, which is calculated by combining serum PSA levels and urine UCA1 expression.

For detecting prostate cancer, the UCA1 score in the Shengli training cohort was obviously superior to the above mentioned PCA3 score and urine epiCaPture, which is a six‐gene DNA methylation panel.^32^ The PCA3 score did not demonstrate an AUC value at a cutoff of >35 and had a sensitivity of only 0.42 at PCA3 score >60.^7^ Similarly, in the epiCaPture cohort study, Eve O'Reilly et al. showed that the AUC of urine epiCaPture for detecting prostate cancer was 0.64.^32^ In this cohort, the UCA1 score performed well (AUC 0.880) and had optimal sensitivity and specificity (0.765 and 0.870) at a cutoff value of −0.475. In addition, DCA also supported that the net profit of the UCA1 score is superior to that of PSA plus DRE and other tools in prostate biopsy determination, because the amount of exfoliated prostate cells in urine sediment varies widely among individuals. Moreover, the expression of UCA1 may be affected by bladder cancer cells.^19^ We used the expression levels of the prostate‐specific marker gene KLK2 in urine to correct the UCA1 score at first consideration. Compared with PCA3 and epiCaPture, UCA1 scores normalized to KLK2 may result in better performance in prostate cancer patients.

Compared with SelectMDx, another well‐known urine marker study for prostate cancer,^34^ our Shengli training cohort showed that the UCA1 score had a better AUC (0.880 vs. 0.76) and PPV (0.67 vs. 0.27). Importantly, SelectMDx is suitable for the detection of high‐grade prostate cancer (Gleason score ≥7). However, the UCA1 score can not only distinguish benign tissue from high‐risk cancer well, but can also distinguish benign tissue from nonhigh‐risk cancer. The UCA1 score of benign patients was significantly lower than that of nonhigh‐risk and high‐risk prostate cancer patients. Specifically, the UCA1 score may be of great diagnostic significance for suspected prostate cancer patients with low or intermediate PSA levels. In patients with PSA <4 (who may be very low‐risk cancer patients), all cancer patients were identified with a PPV of 50% and an NPV of 100% by using UCA1 score ≥−0.475. Currently Daniel W. Kim et al. ^35^ reported that men with a biopsy Gleason score of 9 to 10 and a PSA level of ≤4 versus >4, there was a higher rate of prostate cancer‐specific mortality because they may have pathologic or genetic variants that make them less amenable to a cure with current standards of care. Therefore, accurate detection of prostate cancer patients with low PSA value has important clinical significance. In patients with 4 ≤ PAS < 10 (who may be low‐risk cancer patients) or 10 ≤ PSA < 20 (who may be intermediate‐risk cancer patients), the PPV and NPV were also good. In addition, the relationship between UCA1 score and two widely used risk prediction systems (D'Amico and CAPRA) was investigated by ROC curve analysis. The UCA1 score was better than urine UCA1 gene expression alone or serum PSA levels.

Our results based on the Shengli training cohort supported the notion that the application of the UCA1 score may sharply reduce the burden of prostate biopsy caused by underdiagnosis. However, we also found that in patients with PSA ≥4, the application of UCA1 score ≥−0.475 reduced the biopsy rate and resulted in a higher rate of missed diagnosis. The effects of different numbers of prostate cells present in urine from different individuals were excluded. The possible reason for the high rate of missed diagnosis is that each PCR amplification did not use uniform negative and positive controls for quality control. Therefore, in order to achieve the balance between reduction of biopsy and missed diagnosis, a cutoff value of UCA1 score ≥−3.47 was applied to prostate biopsy decision‐making when PSA ≥4. Most importantly, the performance of the UCA score was verified again in the Fuzhou validation cohort.

A recent study in NEJM (link below) has shown that using biparametric MRI that biopsy rate was halved without missing clinically significant prostate cancer^28^. In our study some patients did not undergo MRI examination before prostate biopsy due to economic reasons. Therefore, UCA1 score and MRI cannot be combined for analysis. In addition, urine UCA1 detection is less expensive than MRI and may be suitable for large‐scale screening and therapeutic monitoring, as well as for remote areas where MRI is not available. Our next study of UCA1 score combined with MRI is necessary for the diagnosis of prostate cancer.

In all patients (*n* = 897) we found that UCA1 score was superior to PSA only for detection of clinically insignificant, clinically significant, group 3 + 4 + 5 of WHO grade groups (Gleason score ≥4 + 3), and high‐risk prostate cancer with higher percentage. Finaly, we analyzed patients without elevated PSA (<4) by ROC and found that UCA1 score had a significantly higher AUC than PSA only without missed diagnosis of prostate cancer. Our next step forward a large population screening including urine UCA1, blood TPSA, and MRI and validate the diagnostic utility and accuracy of the UCA1 score in the men without ANY high‐risk features (PSA or otherwise).

In conclusion, we systematically determined and validated that a novel UCA1 score could serve as a new noninvasive test for early detection and risk prediction of prostate cancer. This is the first urine biomarker study using KLK2 for normalization. The performance of the UCA1 score is superior to that of PSA levels or other existing urine biomarkers. The UCA1 score, which has high sensitivity and could greatly reduce the burden of biopsy, could be used in conjunction with PSA levels and existing tools to assist in decision‐making for prostate diagnosis biopsy.

## CONFLICT OF INTEREST

The author(s) declared no potential conflict of interest with respect to the research, authorship, and/or publication of this article.

## AUTHOR CONTRIBUTIONS

Conception and design: Youdong Lin, Chun Liu. Collection and assembly of data: All authors. Data analysis and interpretation: Youdong Lin, Guihua Liu. Manuscript writing: All authors. Accountable for all aspects of the work: All authors.

## ETHICS STATEMENT

This study was approved by the Medical Ethics Committee of Shengli Clinical Medical College of Fujian Medical University and Fuzhou NO. 1 Hospital Affiliated with Fujian Medical University. All patients signed written informed consent.

## Supporting information


Figure S1

Figure S2

Figure S3

Table S1

Table S2

Table S3

Table S4

Data S1

Data S2

## Data Availability

The data are available in article supplementary material.

## References

[1] U.S. Preventive Servi ces Task Force . Final Recommendation statement:prostate cancer: Screening. Accessed 2021. https://www.uspreventiveservicestaskforce.org/Page/Document/RecommendationStatementFinal/prostate‐cancer‐screening

[2] Carter HB , Albertsen PC , Barry MJ , et al. Early detection of prostate cancer: AUA guideline. J Urol. 2013;190:419‐426. 23659877 10.1016/j.juro.2013.04.119 PMC4020420

[3] Kawachi MH , Bahnson RR , Barry M , et al. NCCN clinical practice guidelines in oncology: prostate cancer early detection. J Natl Compr Canc Netw. 2010;8:240‐262. 20141680 10.6004/jnccn.2010.0016

[4] Heidenreich A , Abrahamsson PA , Artibani W , et al. Early detection of prostate cancer: European Association of Urology recommendation. Eur Urol. 2013;64:347‐354. 23856038 10.1016/j.eururo.2013.06.051

[5] Qaseem A , Barry MJ , Denberg TD , Owens DK , Shekelle P . Clinical guidelines Committee of the American College of physicians. Screening for prostate cancer: a guidance statement from the clinical guidelines Committee of the American College of physicians. Ann Intern Med. 2013;158:761‐769. 23567643 10.7326/0003-4819-158-10-201305210-00633

[6] Smith RA , Andrews K , Brooks D , et al. Cancer screening in the United States, 2016: a review of current American Cancer Society guidelines and current issues in cancer screening. CA Cancer J Clin. 2016;66:96‐114. 26797525 10.3322/caac.21336

[7] Wei JT , Feng Z , Partin AW , et al. Can urinary PCA3 supplement PSA in the early detection of prostate cancer? J Clin Oncol. 2014;32:4066‐4072. 25385735 10.1200/JCO.2013.52.8505 PMC4265117

[8] Catalona WJ , Partin AW , Sanda MG , et al. A multicenter study of [‐2]pro‐prostate specific antigen combined with prostate specific antigen and free prostate specific antigen for prostate cancer detection in the 2.0 to 10.0 ng/ml prostate specific antigen range. J Urol. 2011;185:1650‐1655. 21419439 10.1016/j.juro.2010.12.032 PMC3140702

[9] Parekh DJ , Punnen S , Sjoberg DD , et al. A multi‐institutional prospective trial in the USA confirms that the 4Kscore accurately identifies men with high‐grade prostate cancer. Eur Urol. 2015;68:464‐470. 25454615 10.1016/j.eururo.2014.10.021

[10] Van Neste L , Hendriks RJ , Dijkstra S , et al. Detection of high‐grade prostate cancer using a urine molecular biomarker‐based risk score. Eur Urol. 2016;70:740‐748. 27108162 10.1016/j.eururo.2016.04.012

[11] Tomlins SA , Day JR , Lonigro RJ , et al. Urine TMPRSS2:ERG plus PCA3 for individualized prostate cancer risk assessment. Eur Urol. 2016;70:45‐53. 25985884 10.1016/j.eururo.2015.04.039 PMC4644724

[12] McGinley KF , McMahon GC , Brown GA . Impact of the US preventive services task force grade D recommendation: assessment of evaluations for elevated prostate‐specific antigen and prostate biopsy in a large urology group practice following statement revision. Rev Urol. 2015;17:171‐177. 26543432 PMC4633661

[13] Siddiqui MM , Rais‐Bahrami S , Turkbey B , et al. Comparison of MR/ ultrasound fusion‐guided biopsy with ultrasound‐guided biopsy for the diagnosis of prostate cancer. Jama. 2015;313:390‐397. 25626035 10.1001/jama.2014.17942 PMC4572575

[14] Barrett T , Haider MA . The emerging role of MRI in prostate cancer active surveillance and ongoing challenges. AJR Am J Roentgenol. 2017;208:131‐139. 27726415 10.2214/AJR.16.16355

[15] Ahmed HU , El‐Shater Bosaily A , Brown LC , et al. Diagnostic accuracy of multi‐parametric MRI and TRUS biopsy in prostate cancer (PROMIS): a paired validating confirmatory study. Lancet. 2017;389:815‐822. 28110982 10.1016/S0140-6736(16)32401-1

[16] Yu Y , GaoYu Y , Gao F , He Q , Li G , Ding G . lncRNA UCA1 functions as a ceRNA to promote prostate cancer progression via sponging miR143. Mol Ther Nucleic Acids. 2020;19:751‐758. 31954329 10.1016/j.omtn.2019.11.021 PMC6962633

[17] He C , Lu X , Yang F , et al. LncRNA UCA1 acts as a sponge of miR‐204 to up‐regulate CXCR4 expression and promote prostate cancer progression. Biosci Rep. 2019;39:BSR20181465. 30940776 10.1042/BSR20181465 PMC6499452

[18] Zhao X , Wang Y , He J , et al. LncRNA UCA1 maintains the low‐tumorigenic and nonmetastatic status by stabilizing E‐cadherin in primary prostate cancer cells. Mol Carcinog. 2020;59:1174‐1187. 32805084 10.1002/mc.23247

[19] Xue M , Chen W , Xiang A , et al. Hypoxic exosomes facilitate bladder tumor growth and development through transferring long non‐coding RNA‐UCA1. Mol Cancer. 2017;16:143. 28841829 10.1186/s12943-017-0714-8 PMC5574139

[20] D'Amico AV , Whittington R , Malkowicz SB , et al. Biochemical outcome after radical prostatectomy, external beam radiation therapy, or interstitial radiation therapy for clinically localized prostate cancer. Jama. 1998;280:969‐974. 9749478 10.1001/jama.280.11.969

[21] Cooperberg MR , Pasta DJ , Elkin EP , et al. The University of California, san Francisco cancer of the prostate risk assessment score: a straightforward and reliable preoperative predictor of disease recurrence after radical prostatectomy. J Urol. 2005;173:1938‐1942. 15879786 10.1097/01.ju.0000158155.33890.e7 PMC2948569

[22] O'Reilly E , Tuzova AV , Walsh AL , et al. epiCaPture: a urine DNA methylation test for early detection of aggressive prostate cancer. JCO Precis Oncologia. 2019;3:1‐8. 10.1200/PO.18.00134 PMC6383793 30801051

[23] Carroll PR , Parsons JK , Andriole G , et al. NCCN guidelines insights: prostate cancer early detection, version 2.2016. J Natl Compr Canc Netw. 2016;14:509‐519. 27160230 10.6004/jnccn.2016.0060 PMC10184498

[24] Groskopf J , Aubin SM , Deras IL , et al. APTIMA PCA3 molecular urine test: development of a method to aid in the diagnosis of prostate cancer. Clin Chem. 2006;52:1089‐1095. 16627561 10.1373/clinchem.2005.063289

[25] Bustin SA , Benes V , Garson JA , et al. The MIQE guidelines: minimum information for publication of quantitative real‐time PCR experiments. Clin Chem. 2009;55:611‐622. 19246619 10.1373/clinchem.2008.112797

[26] Riley RD , Snell KI , Ensor J , et al. Minimum sample size for developing a multivariable prediction model: PART II ‐ binary and time‐to‐event outcomes. Stat Med. 2019;38:1276‐1296. 30357870 10.1002/sim.7992 PMC6519266

[27] Kimura T , Egawa S . Epidemiology of prostate cancer in Asian countries. Int J Urol. 2018;25:524‐531. 29740894 10.1111/iju.13593

[28] Eklund M , Jäderling F , Discacciati A , et al. MRI‐targeted or standard biopsy in prostate cancer screening. N Engl J Med. 2021;385:908‐920. 34237810 10.1056/NEJMoa2100852

[29] Schoots IG , Roobol MJ , Nieboer D , Bangma CH , Steyerberg EW , Hunink MGM . Magnetic resonance imaging‐targeted biopsy may enhance the diagnostic accuracy of significant prostate cancer detection compared to standard transrectal ultrasound‐guided biopsy: a systematic review and meta‐analysis. Eur Urol. 2015;68:438‐450. 25480312 10.1016/j.eururo.2014.11.037

[30] Heijnsdijk EA , Wever EM , Auvinen A , et al. Quality‐of‐life effects of prostate specific antigen screening. N Engl J Med. 2012;367:595‐605. 22894572 10.1056/NEJMoa1201637 PMC4982868

[31] Andriole GL . Pathology: the lottery of conventional prostate biopsy. Nat Rev Urol. 2009;6:188‐189. 19352393 10.1038/nrurol.2009.46

[32] Faiena I , Holden S , Cooperberg MR , et al. Goldilocks principle: how much is just right? J Clin Oncol. 2018;36:937‐941. 29401003 10.1200/JCO.2017.76.4050 PMC6804825

[33] Carroll PH , Mohler JL . NCCN guidelines updates: prostate cancer and prostate cancer early detection. J Natl Compr Canc Netw. 2018;16:620‐623. 29784740 10.6004/jnccn.2018.0036

[34] Leyten GH , Hessels D , Smit FP , et al. Identification of a candidate gene panel for the early diagnosis of prostate cancer. Clin Cancer Res. 2015;21:3061‐3070. 25788493 10.1158/1078-0432.CCR-14-3334

[35] Kim DW , Chen MH , Wu J , et al. Prostate‐specific antigen levels of ≤4 and >4 ng/mL and risk of prostate cancer‐specific mortality in men with biopsy Gleason score 9 to 10 prostate cancer. Cancer. 2021;127:2222‐2228. 34101827 10.1002/cncr.33503

