# Supplementary figures and images for: Urothelial carcinoembryonic antigen 1 score for early detection of prostate cancer and risk prediction
Source: Cancer Med. 2022 Mar 15;11(15):2875–85. doi: 10.1002/cam4.4629 (PMC9359874; doi:10.1002/cam4.4629)

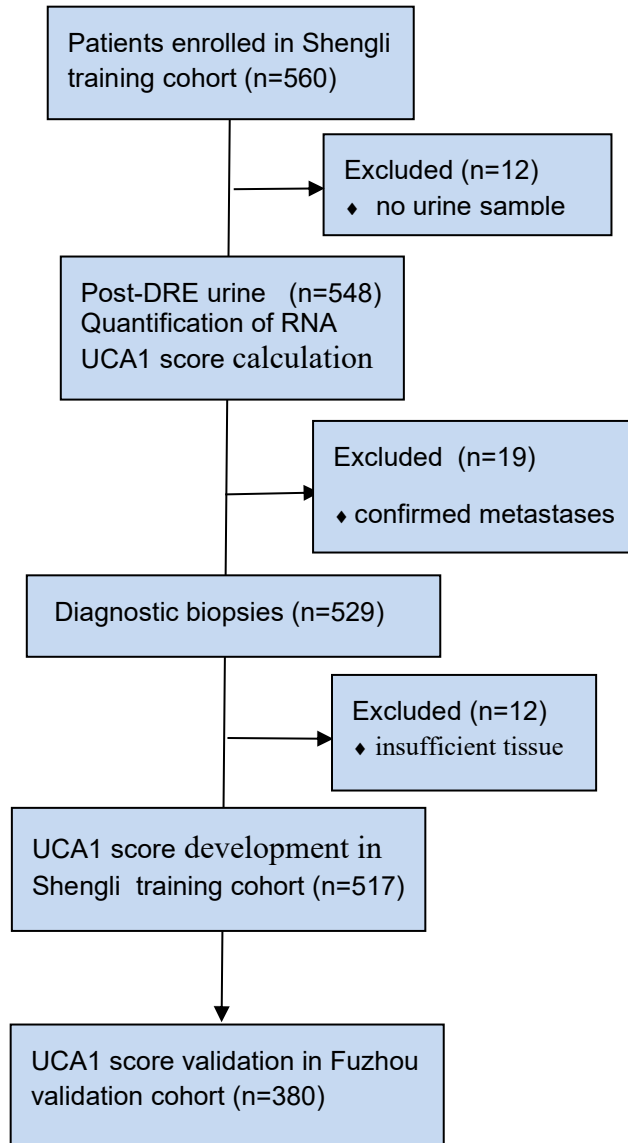

Supplement: Supplementary file 1 — Figure S1 [file CAM4-11-2875-s005.pdf]

# ROC for detecting cancer in men without elevated PSA (<4)

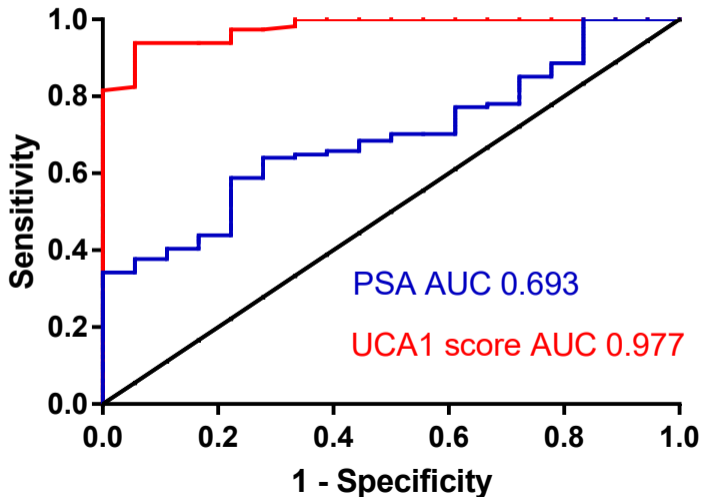

Supplement: Supplementary file 3 — Figure S3 [file CAM4-11-2875-s001.pdf]
