# Supplementary material for: Urothelial carcinoembryonic antigen 1 score for early detection of prostate cancer and risk prediction
Source: Cancer Med. 2022 Mar 15;11(15):2875–85. doi: 10.1002/cam4.4629 (PMC9359874; doi:10.1002/cam4.4629)

Figure S2 Difference in detection of clinically significant cancer and high-risk cancer

(two-sided 95% CI)

percentage points

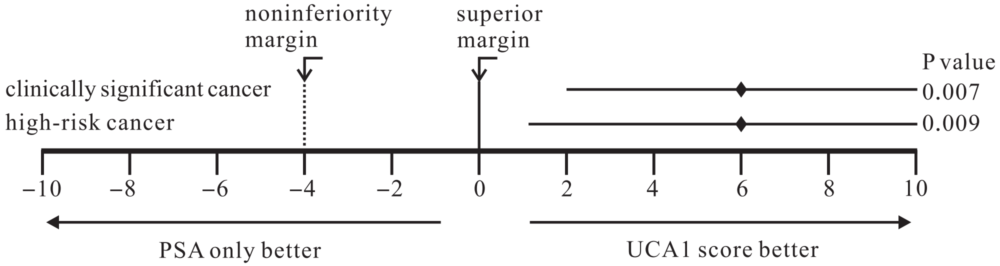

Supplement: Supplementary file 2 — Figure S2 [file CAM4-11-2875-s008.pdf]
